# Model-based prediction of fusimotor activity during active wrist movements

**DOI:** 10.1186/1471-2202-14-S1-O16

**Published:** 2013-07-08

**Authors:** Bernard Grandjean, Marc A Maier

**Affiliations:** 1 CNRS UMR 8194, Université Paris Descartes, Sorbonne Paris Cité, Paris, F-75006, France; 2 Univ Paris Diderot, Sorbonne Paris Cité, Paris, F-75013, France

## Introduction

Muscle spindles, whose activity is determined by muscle length changes and by fusimotor drive (i.e. γ-drive), provide critical information about movement position and velocity [1]. However, task-dependent fusimotor drive remains largely unknown [2], since no fusimotor neurons have ever been recorded during active, voluntary upper limb movements, whether in animals nor in humans. So far an estimation of γ-drive could only be obtained through an indirect inference of fusimotor activity from observed muscle spindle activity. Our aim was to model the effect of γ-drive on muscle spindles and to simulate voluntary wrist movements for which the spindle responses are empirically known.

## Methods

**Figure 1:**
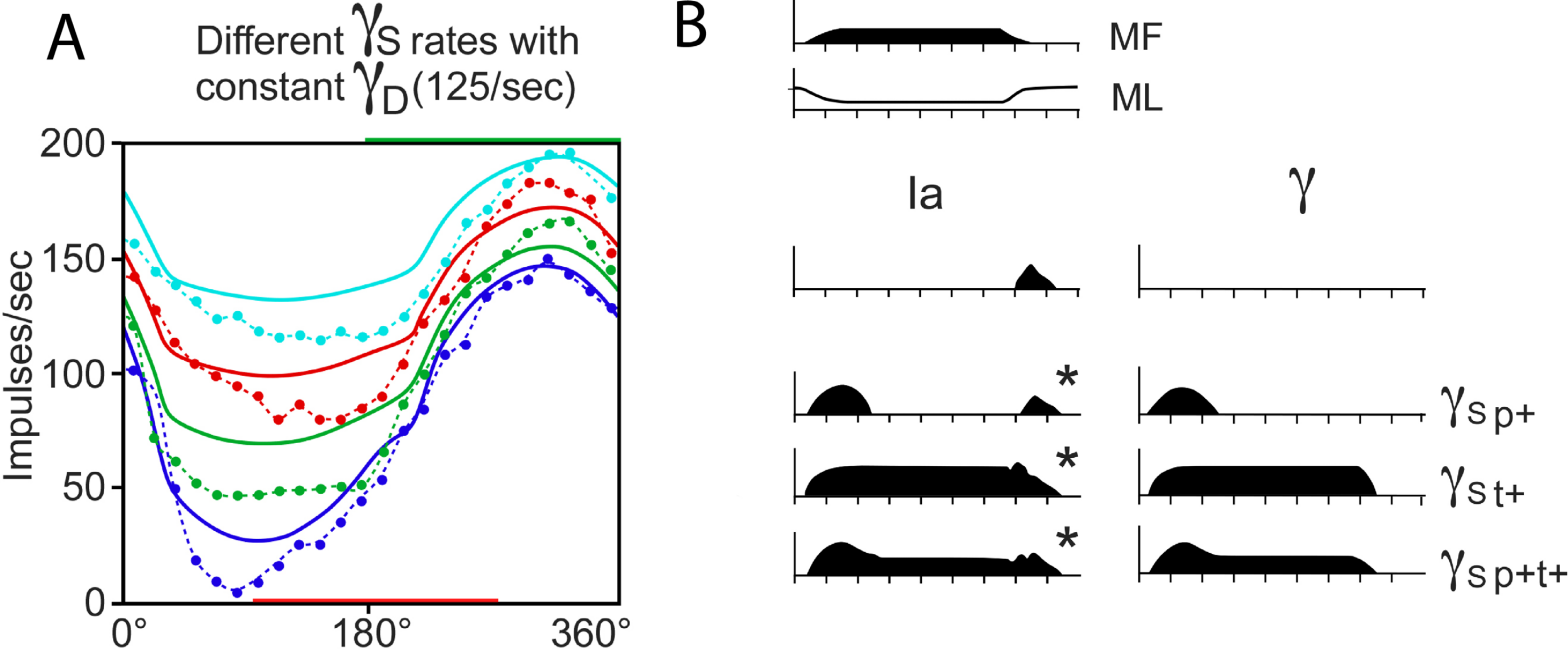
**A. Fit between passive **[4]**(dotted lines) and simulated (lines) Ia responses during sinusoidal stretch under constant γ_D_-drive (125 Hz) and 4 different rates of γ_S_-drive (top to bottom: 125, 75, 50, 0 Hz)**. B. Simulated Ia responses (left column) during active muscle contraction for 4 different γ_S_-drives (right column): no, phasic, tonic and phasic-tonic drive. * indicates simulated responses similar to empirically observed Ia responses [5].

Our conceptually simple computational model (an adaptation of [3]) allows for a direct quantification of γ-drive. A forward calculation predicts spindle responses based on time-varying γ-drive and muscle length changes. This computational model thus links a biomechanical (musculo-tendon) wrist model to length- and γ-drive-dependent transfer functions of group Ia and group II muscle spindles. These transfer functions were calibrated (Figure 1A) with extant data from passive movements in the cat [4].

## Results

Our simulations suggest that (i) empirically observed muscle spindle activity profiles can to a large part be explained by a strongly task-dependent γ-drive (Figure 1B), (ii) observed differences between individual muscle spindle response profiles can be explained by a corresponding variability in the γ-drive (Figure 1B), and (iii) observed phase advance of spindle responses can to a large part be explained by appropriate γ-drive.

## Conclusion

Our simulation predicts that γ-drive is strongly modulated and task-dependent and that appropriate γ-drive can explain many empirically observed aspects of group Ia and II muscle spindle responses during active movements.
